# Genetic Diversity and Spatial Genetic Structure of the Grassland Perennial *Saxifraga granulata* along Two River Systems

**DOI:** 10.1371/journal.pone.0130463

**Published:** 2015-06-16

**Authors:** Sascha van der Meer, Hans Jacquemyn

**Affiliations:** KU Leuven, Laboratory of Plant Conservation and Population Biology, Kasteelpark Arenberg 31- bus 02435, Heverlee, Belgium; Wuhan Botanical Garden,CAS, CHINA

## Abstract

Due to changes in land use, the natural habitats of an increasing number of plant species have become more and more fragmented. In landscapes that consist of patches of suitable habitat, the frequency and extent of long-distance seed dispersal can be expected to be an important factor determining local genetic diversity and regional population structure of the remaining populations. In plant species that are restricted to riparian habitats, rivers can be expected to have a strong impact on the dynamics and spatial genetic structure of populations as they may enable long-distance seed dispersal and thus maintain gene flow between fragmented populations. In this study, we used polymorphic microsatellite markers to investigate the genetic diversity and the spatial genetic structure of 28 populations of *Saxifraga granulata* along two rivers in central Belgium. We hypothesized that rivers might be essential for gene flow among increasingly isolated populations of this species. Genetic diversity was high (*H_S_* = 0.68), which to a certain extent can be explained by the octoploid nature of *S*. *granulata* in the study area. Populations along the Dijle and Demer rivers were also highly differentiated (*G”*_ST_ = 0.269 and 0.164 and *D*_EST_ = 0.190 and 0.124, respectively) and showed significant isolation-by-distance, indicating moderate levels of gene flow primarily between populations that are geographically close to each other. Along the river Demer population genetic diversity was higher upstream than downstream, suggesting that seed dispersal via the water was not the primary mode of dispersal. Overall, these results indicate that despite increasing fragmentation populations along both rivers were highly genetically diverse. The high ploidy level and longevity of *S*. *granulata* have most likely buffered negative effects of fragmentation on genetic diversity and the spatial genetic structure of populations in riparian grasslands.

## Introduction

Habitat loss and fragmentation decrease the size and increase the spatial isolation of many plant populations in Europe and elsewhere in the world [1,2]. Plant populations rely on ecological and genetic processes that ensure connectivity and meta-population viability. Ensuring pollen or seed flow between remnant habitat patches is therefore vital for species persistence in patchy landscapes as recurrent gene flow affects the rate of population expansion, recruitment, and colonization of new suitable habitat and allows replenishment of alleles that were lost after fragmentation [3–6].

In riparian habitats, rivers can be expected to be important to maintain gene flow between populations. Natural river landscapes consist of a mosaic of habitats that are tightly connected to each other by water movement [7,8]. Rivers within riparian ecosystems are characterized by unidirectional water movement, are generally dynamic and often show large variation in discharge levels after periods of low or heavy rainfall [9]. Gene flow between linearly arranged populations, as seen along rivers, can follow two main patterns; it can occur primarily between adjacent populations, leading to increasing genetic distances with increasing geographical distances (i.e. isolation-by-distance) [10]. Alternatively, gene flow can occur at a similar rate among all populations, even over long distances, leading to low genetic differentiation among populations without an isolation-by-distance effect. Interpretation of the direction and distance of gene flow in relation to measures of isolation-by-distance may be complicated by meta-population dynamics such as extinction and colonization events [11,12]. Founder events, for instance, can increase genetic population differentiation and can cause the relationship between genetic and geographic distances to disappear [13–15]. Furthermore, the direction of gene flow along linearly arranged populations can be unidirectional or bi-directional and gene flow in both directions can occur at a different rate. Moreover, gene flow can occur through both pollen and seeds. When gene flow occurs mainly via pollinators one would expect a bi-directional isolation-by-distance pattern.

Given that river systems can be characterized by unidirectional water movement seed dispersal via the water (i.e. hydrochory) will most likely occur downstream. When downstream migration is slightly higher than upstream migration, genetic diversity could accumulate downstream, while populations upstream may gradually lose genetic diversity and in the most extreme case may disappear due to stochastic events [16]. This phenomenon is known as ‘the unidirectional dispersal hypothesis’ [12,17–19]. However, the observation that upstream habitats along rivers are not depleted of riparian plant species and populations upstream are generally genetically diverse as well, indicates that species are capable of dispersing seeds, pollen or vegetative structures upstream as well as downstream [11,20,21]. For instance, riparian populations of *Sparganium emersum* and *Populus nigra* disperse upstream via birds and wind, while downstream migration might have occurred via river systems [22,23].

The occurrence and frequency of long-distance seed dispersal via rivers also depends on the distance to the streamline. Several studies of riparian plant species have shown that there is a clear relationship between distance to the river and population genetic differentiation [12,19]. Some riparian plant species grow right at the waterfront, which enables them to drop seeds directly into the water. Grassland populations that are situated further from the river might still depend on river dynamics to ensure connectivity between populations, for instance, through enabling seed dispersal over long distances during seasonal or less frequent flooding events [23,24]. However, construction of dams and dikes along rivers can diminish connectivity between riparian grassland populations [25].

The aim of this study was to assess whether rivers shape the genetic diversity and spatial genetic structure of fragmented populations of the grassland perennial *Saxifraga granulata* along two rivers in Belgium. Over the last decades, populations of *S*. *granulata* have become smaller and more isolated due to habitat loss and fragmentation [26,27]. The species used to occur in dry to mesic grasslands all over Western Europe. Due to intensification of agricultural practices the majority of populations in the study area is now restricted to riparian meadows [26]. Hence, rivers can be expected to be important in ensuring connectivity among populations of this species and therefore in maintaining genetic diversity. To test this hypothesis, we used highly polymorphic microsatellite markers to study the spatial genetic structure of *S*. *granulata* along two dynamic river systems. More specifically, we asked the following questions:

1. Are populations of *S*. *granulata* genetically differentiated within both river systems?
2. Does gene flow mainly occur between populations that are geographically close to each other?
3. Do we find accumulation of genetic diversity downstream, indicating that the river is the main dispersal vector?

## Material and Methods

### Study species

*S*. *granulata*, meadow saxifrage, is a self-compatible, insect-pollinated, protandrous, perennial rosette herb [28]. The species can reproduce sexually as well as clonally by formation of small bulbils at the base of the plant [29]. Individual ramets flower in May and June, producing flowering stems of up to 57 cm in height with small white flowers that are pollinated predominantly by unspecialized flies and solitary bees [30]. Populations in Western Europe have shown a decline during the last decades. In our study area the species is also listed as ‘declining’ [31], mainly as a result of the increasing use of fertilizers in meadows and agricultural landscapes [26,27].

### Study area and population sampling

**Fig 1:**
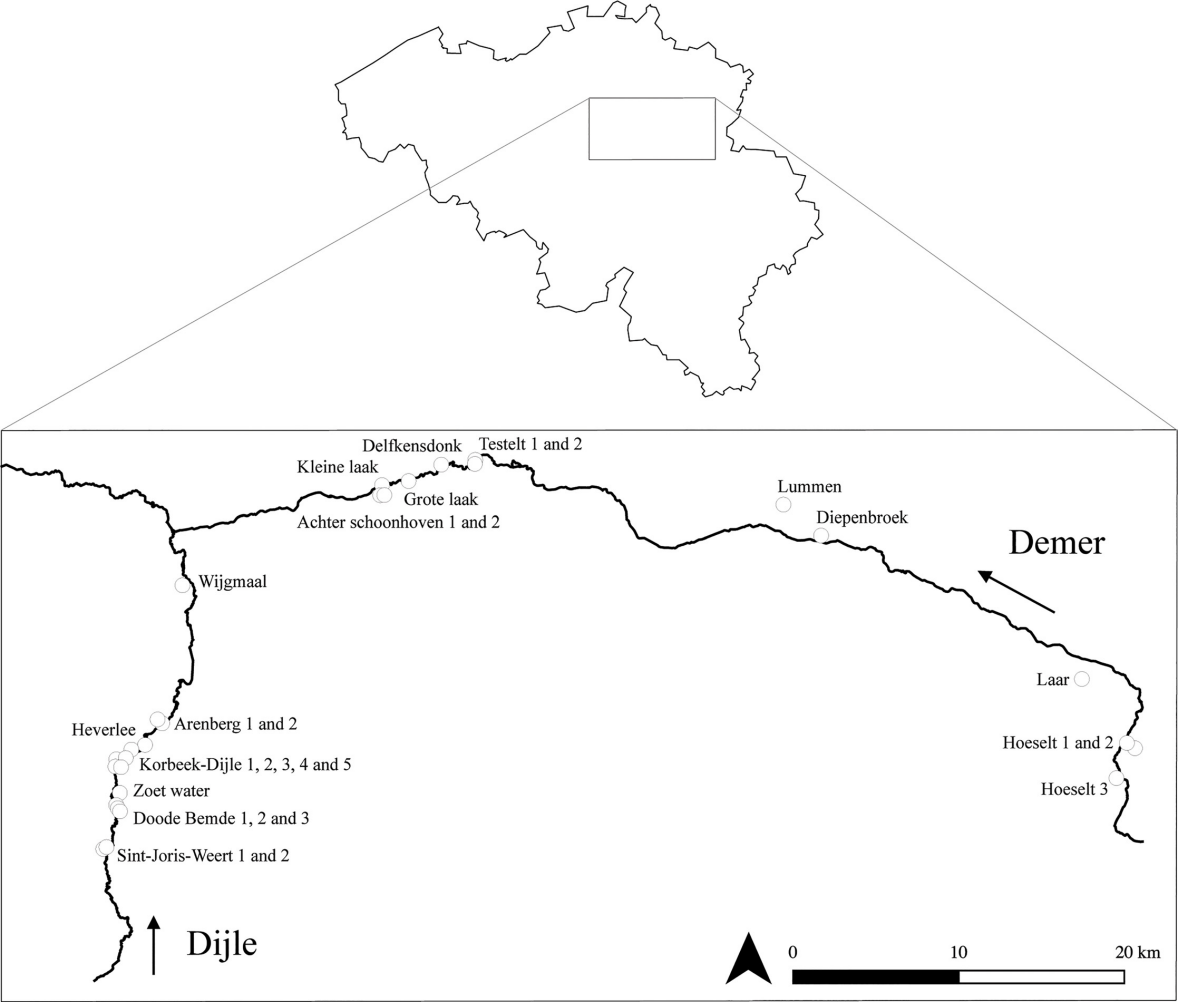
Study area in Belgium and locations of the 28 sampling sites along the rivers Dijle and Demer. The arrows indicate the direction of the water flow. The figure was constructed based on maps of the NGI (Nationaal Geographisch Instituut) using QGIS 2.6.0 [65].

The study was conducted in the central part of Belgium (Fig 1), where *S*. *granulata* used to be a widespread species in the first half of 20th century, but nowadays is confined to riparian grasslands. The study was conducted along the rivers Dijle and Demer (Fig 1), which are both about 90 km long, dynamic, rain-fed rivers, with soft riversides. Both rivers can reach high discharge levels after heavy rainfall, but have a low discharge during dry periods. Alongside both rivers are valleys with mesic to wet grasslands that are well-connected to the river systems. Both rivers regularly flooded and inundated the adjacent river valleys in the past [32–34]. Nowadays, large-scale flooding events that affected the whole study area roughly take place once every ten years. Along the river Demer the last large-scale flooding events were in 1998 and 2002 [34], while populations along the Dijle were flooded in 2010 [35]. Small-scale flooding events are common along the river Dijle [35], but are less frequent along the Demer river [34].

During the flowering season of 2012 we visited 15 populations of *S*. *granulata* along the Dijle River and 13 populations along the Demer River (Fig 1). Sampled populations were situated at least 200 m and at most 47 km apart within both river systems (Dijle: mean distance = 4.4 km, SD = 3.7; Demer: mean distance = 23.6 km, SD = 17.7). Differences in elevation of the populations were very small as they were all situated on the floodplains of the rivers. We tried to sample all populations that were situated within 5 km of the studied river systems using information from local reserve managers and the website ‘waarnemingen.be’ [36].

In each population we collected leaf material from 20 individuals and we sampled individuals that were at least one meter apart to avoid collecting clones. Fresh leaf material was immediately dried in silica gel prior to DNA extraction. We estimated population size by counting the total number of flowering ramets and measured the average distance to the river in each population (Table 1).

**Table 1:** Characteristics of the studied *Saxifraga granulata* populations along the river systems Dijle and Demer.

	Population	Abbr.	Distance to river (m)	Pop. size	Mean nr. of alleles	Mean effective nr. of alleles	*H*_S_	*H*_T_
Dijle	Sint-Joris-Weert 1	sjw1	230	104	6.4	3.9	0.66	
	Sint-Joris-Weert 2	sjw2	200	179	5.4	3.2	0.51	
	Doode Bemde 1*	db1	130	490	6.8	3.7	0.64	
	Doode Bemde 2*	db2	10	1000	6.4	3.6	0.61	
	Doode Bemde 3*	db3	10	242	5.0	2.8	0.61	
	Zoet Water*	zw	230	1000	6.0	3.4	0.66	
	Korbeek-Dijle 1	kd1	30	180	7.3	4.4	0.73	
	Korbeek-Dijle 2	kd2	50	206	6.6	3.5	0.68	
	Korbeek-Dijle 3	kd3	300	650	6.1	3.5	0.67	
	Korbeek-Dijle 4	kd4	30	136	6.6	3.9	0.63	
	Korbeek-Dijle 5	kd5	140	230	6.8	4.2	0.67	
	Heverlee	he	20	321	5.3	2.5	0.50	
	Arenberg 1	ab1	0	1350	6.9	4.0	0.66	
	Arenberg 2	ab2	150	350	5.2	3.3	0.63	
	Wijgmaal*	wm	500	210	5.9	3.4	0.64	
	Mean (± SD)		135 (± 140)	443 (± 383)	6.2 (± 0.7)	3.6 (±0.5)	0.63 (±0.06)	0.70
Demer	Hoeselt 3	hoe3	160	800	8.4	4.5	0.74	
	Hoeselt 2	hoe2	500	650	8.1	4.7	0.74	
	Hoeselt 1	hoe1	110	800	7.8	4.9	0.76	
	Laar	la	900	1500	7.7	4.4	0.75	
	Diepenbroek	dpb	250	94	7.8	4.3	0.71	
	Lummen	lu	980	800	7.1	3.9	0.68	
	Testelt 1	t1	140	250	6.7	3.6	0.69	
	Testelt 2	t2	20	500	6.7	3.7	0.68	
	Delfkensdonk*	dd	140	800	7.9	4.1	0.73	
	Grote Laak*	gl	10	930	7.3	4.2	0.74	
	Kleine Laak*	kl	10	50	7.7	4.1	0.72	
	Achter Schoonhoven 2*	sh2	400	5000	8.1	4.4	0.70	
	Achter Schoonhoven 1*	sh1	400	220	7.2	4.0	0.68	
	Mean (± SD)		309 (± 321)	953 (± 1280)	7.6 (± 0.5)	4.2 (± 0.4)	0.72 (± 0.03)	0.75

Populations are ordered according to their position along the river, the upper most population in the table occurs most upstream.

*H*_S_ is the heterozygosity within populations, *H*_T_ the total heterozygosity for both river systems.

* Sampling locations situated in grasslands managed by ‘Natuurpunt’.

*S*. *granulata* is not protected in Belgium, therefore, no collection permit was required. However, we did collect leaf material in nature reserves managed by the nature protection NGO Natuurpunt, this concerns populations ‘Doode Bemde 1, 2 and 3’, ‘Zoet Water’, ‘Wijgmaal’, ‘Achter Schoonhoven 1 and 2’, ‘Kleine Laak’, ‘Grote Laak’ and ‘Delfkensdonk’. Natuurpunt granted us permission to work in these grasslands. All other populations were located outside protected areas and were not situated on private land, hence, no specific permission was required for working in these populations.

### DNA extraction and microsatellite analysis

For DNA extraction circa 20 mg of dried plant material was homogenized to a fine powder using the mixer mill MM 200 (Retsch, Haan, Germany) and two small ceramic beads. DNA was extracted from a total of 560 plants following the NucleoSpin Plant II protocol for genomic DNA (Macherey-Nagel, Düren, Germany). For cell lysis we used buffer PL1 and incubated the cell lysis suspension for 60 min. DNA concentration and quality were measured using a NanoDrop ND-2000 spectrophotometer (Thermo Scientific, Wilmington, DE, USA).

Nine recently developed microsatellite markers were used for genetic analysis. In a previous study these markers indicated that *S*. *granulata* is octoploid in our study area [37]. All loci contained at least three and a maximum of eight alleles per genotype. As the microsatellite profiles showed no differentiation of allele sets and allele combinations were completely random (no fixed heterozygosity) *S*. *granulata* is presumably an auto- rather than an allopolyploid [38].

The markers were amplified in two separate PCR multiplexes in a 2720 Thermal Cycler (Applied Biosystems, CA, USA). The total PCR multiplex reaction volume was 10 μl; containing 5 μl Qiagen Multiplex PCR Master Mix (Qiagen, Hilden, Germany), 3 μl RNAse-free water, 1 μl of one of the two multiplexed primer combinations (25 ng/μl) and 1 μl of template DNA (50 ng/μl). Both PCR multiplexes followed the same thermocycler program with initial denaturation of 15 min at 95°C; 27 cycles of 30 sec at 95°C, 1.5 min at 58°C and 1 min at 72°C; and a final elongation of 30 min at 60°C. Then, 1 μl of the PCR reaction was added to a solution of 8.8 μl formamide and 0.2 μl of GeneScan 500 LIZ (-35; -250) size standard (Applied Biosystems). Fragments were sized on an ABI Prism and analyzed by capillary electrophoresis using the 3130 Genetic Analyzer (Applied Biosystems). Samples from all populations were randomly distributed across the ABI plates to prevent influence of gel-artifacts in the data. The raw genetic data were scored using GeneMapper Software v4.0 (Applied Biosystems) using the default settings for microsatellites. Panels and bins for GeneMapper were manually constructed and all data were visually checked to make sure that the loci were identified correctly.

### Data analysis

Because of the octoploid nature of *S*. *granulata* in the study area, population genetic data were analyzed using the program GenoDive 2.0 [39] and the r-package ‘adegenet’ 1.3–6 [40] in R 2.15.1 [41]. Three measures of genetic diversity [42] were calculated for each population using the program GenoDive: the number of alleles, the effective number of alleles (i.e. the number of alleles in a population weighted by their frequencies), and the so-called ‘gametic heterozygosity’ (*H*_S_) [43], which is equivalent to the expected heterozygosity (*H*_*E*_) in diploid species [44]. Analyses were corrected for unknown dosage of alleles, based on the method of [45], since it is hard to estimate allele dosage in octoploid individuals.

Several measures of population differentiation; *G*_ST_, *G”*_ST_ [44] and *D*_EST_ [46], were calculated in GenoDive to assess the genetic structure among populations along both river systems. These population differentiation statistics have their specific advantages and disadvantages. *G*_ST_ (*F*_ST_ analogue), for instance, relates the amount of genetic variation among populations to the total genetic variation over all populations and is determined by the amount of within-population diversity (*H*_S_). Hence, *G*_ST_ = (*H*_T_−*H*_S_)/*H*_T_ [42]. However, when within-population diversity is high, as can be expected in polyploid individuals analyzed with multi-allelic markers, *G*_ST_ may be underestimated. For this reason, we also calculated *G”*_ST_ and *D*_EST_, which have the advantage that they are not negatively dependent on the amount of within-population diversity [47]. To assess the significance of the obtained population differentiation statistics we performed 9999 permutations and we corrected for unknown dosage of alleles. Pairwise population differentiation (*G”*_ST_) values were calculated as well.

For both river systems, the spatial genetic structure of populations was analyzed using a Mantel test in GenoDive and a spatial Principal Component Analysis (sPCA) [48] using the r-package ‘adegenet’. The Mantel test, performed with 9999 permutations, analyzed the relationship between two triangular matrices, one containing pairwise population differentiation values (*G”*_ST_) and the other log-transformed distances. sPCA was used as an alternative spatial analysis method and includes the genetic variation as well as the spatial structure of populations along both rivers. Based on the spatial coordinates a Delaunay triangulation connection network was built between populations and the largest eigenvalues, based on genetic variance and spatial structure, were retained for the analysis [49]. To identify genetic groups of populations within both river systems we performed an AMOVA based K-means clustering analysis in GenoDive [50]. The simulated annealing algorithm was run with 50 000 steps, with correction for unknown dosage of alleles and with 1000 algorithm repeats. The optimal number of clusters was chosen based on pseudo-f scores [51].

To investigate patterns of genetic diversity of *S*. *granulata* along the two rivers, we first related genetic diversity to population size and distance to the river. We expected genetic diversity to increase with increasing population size and that populations closer to the river would be more genetically diverse since the chances of receiving seeds via the water is higher. These relationships were assessed using linear models in R. Finally, we tested the hypothesis that the rivers are the main seed dispersal vectors (i.e. the unidirectional dispersal hypothesis) by relating the position of each population along the river to the population genetic diversity measure ‘number of alleles’, using a linear model in R. Position along the river was quantified as the log transformed distance between populations starting from the first upstream population.

## Results

### Genetic diversity

Populations along the river Demer were significantly more diverse than populations along the Dijle (mean effective number of alleles: *t*_26_ = 4.0, *P* < 0.001 and *H*_S_: t_26_ = 4.8, *P* < 0.001; Table 1). Overall, the number of alleles per locus, for all 560 individuals, ranged from 6 to 21. The total number of alleles per locus within populations ranged from 5 to 8 and the mean effective number of alleles per locus was lower, ranging from 2.50 to 4.87. Within population heterozygosities were high, ranging from 0.50 to 0.76 (Table 1). In general, the most diverse populations were Hoeselt 1 and Laar, two upstream populations along the Demer River. Populations with the lowest genetic diversity were Heverlee and Sint-Joris-Weert 2, two populations along the Dijle River. All multilocus genotypes analyzed were unique, indicating that we did not sample clones.

### Population differentiation

The population differentiation statistic *G*_ST_ was low for both the Dijle and Demer Rivers, 0.093 and 0.042, respectively. The more recently developed population differentiation statistics *G”*_ST_ and *D*_EST_ were higher than *G*_ST_ along both river systems (*G”*_ST_ = 0.269 and 0.164 and D_EST_ = 0.190 and 0.124 for the rivers Dijle and Demer, respectively). All population differentiation statistics were highly significant (*P* < 0.001) for both rivers. Pairwise genetic differentiation values (*G”*_ST_) for populations along the river Dijle ranged from 0.033 to 0.715 with an average of 0.264 (SD = 0.144; Table 2). Population ‘Heverlee’, situated in the middle of the Dijle range, seemed to be most genetically differentiated among the Dijle populations, despite being geographically close to several other populations. Along the Demer, pairwise differentiation values were lower, ranging from 0.002 to 0.312 with an average of 0.152 (SD = 0.066; Table 2).

**Table 2:** Pairwise population differentiation (*G”*_ST_) of *S*. *granulata* along the Dijle and Demer river systems, corrected for unknown dosage of alleles.

	sjw1	sjw2	db1	db2	db3	zw	kd1	kd2	kd3	kd4	kd5	he	ab1	ab2	wm
sjw1	-														
sjw2	0.255	-													
db1	0.256	0.200	-												
db2	0.180	0.038	0.102	-											
db3	0.355	0.338	0.495	0.363	-										
zw	0.241	0.179	0.119	0.108	0.343	-									
kd1	0.100	0.173	0.194	0.109	0.299	0.161	-								
kd2	0.131	0.209	0.200	0.153	0.283	0.181	0.033	-							
kd3	0.148	0.168	0.143	0.138	0.354	0.182	0.097	0.145	-						
kd4	0.091	0.102	0.136	0.108	0.365	0.201	0.116	0.213	0.133	-					
kd5	0.099	0.116	0.135	0.091	0.306	0.193	0.105	0.153	0.131	0.081	-				
he	0.431	0.523	0.433	0.484	0.715	0.472	0.419	0.574	0.364	0.453	0.496	-			
ab1	0.269	0.336	0.176	0.228	0.545	0.334	0.179	0.187	0.233	0.240	0.166	0.460	-		
ab2	0.406	0.329	0.272	0.234	0.540	0.359	0.320	0.369	0.342	0.331	0.197	0.579	0.238	-	
wm	0.265	0.402	0.329	0.254	0.511	0.194	0.240	0.281	0.314	0.340	0.342	0.384	0.394	0.470	-
	hoe3	hoe2	hoe1	la	dpb	lu	t1	t2	dd	gl	kl	sh2	sh1		
hoe3	-														
hoe2	0.083	-													
hoe1	0.124	0.134	-												
la	0.101	0.171	0.098	-											
dpb	0.200	0.171	0.067	0.093	-										
lu	0.196	0.050	0.187	0.267	0.221	-									
t1	0.199	0.183	0.184	0.301	0.258	0.060	-								
t2	0.186	0.147	0.126	0.275	0.260	0.069	0.002	-							
dd	0.090	0.183	0.173	0.175	0.248	0.288	0.312	0.252	-						
gl	0.072	0.123	0.132	0.183	0.195	0.206	0.208	0.196	0.077	-					
kl	0.083	0.071	0.151	0.116	0.131	0.180	0.181	0.197	0.187	0.041	-				
sh2	0.073	0.102	0.110	0.215	0.207	0.109	0.148	0.097	0.206	0.055	0.134	-			
sh1	0.154	0.140	0.187	0.247	0.237	0.140	0.166	0.105	0.206	0.092	0.148	0.005	-		

For abbreviations of population names see Table 1.

### Spatial genetic structure

**Fig 2:**
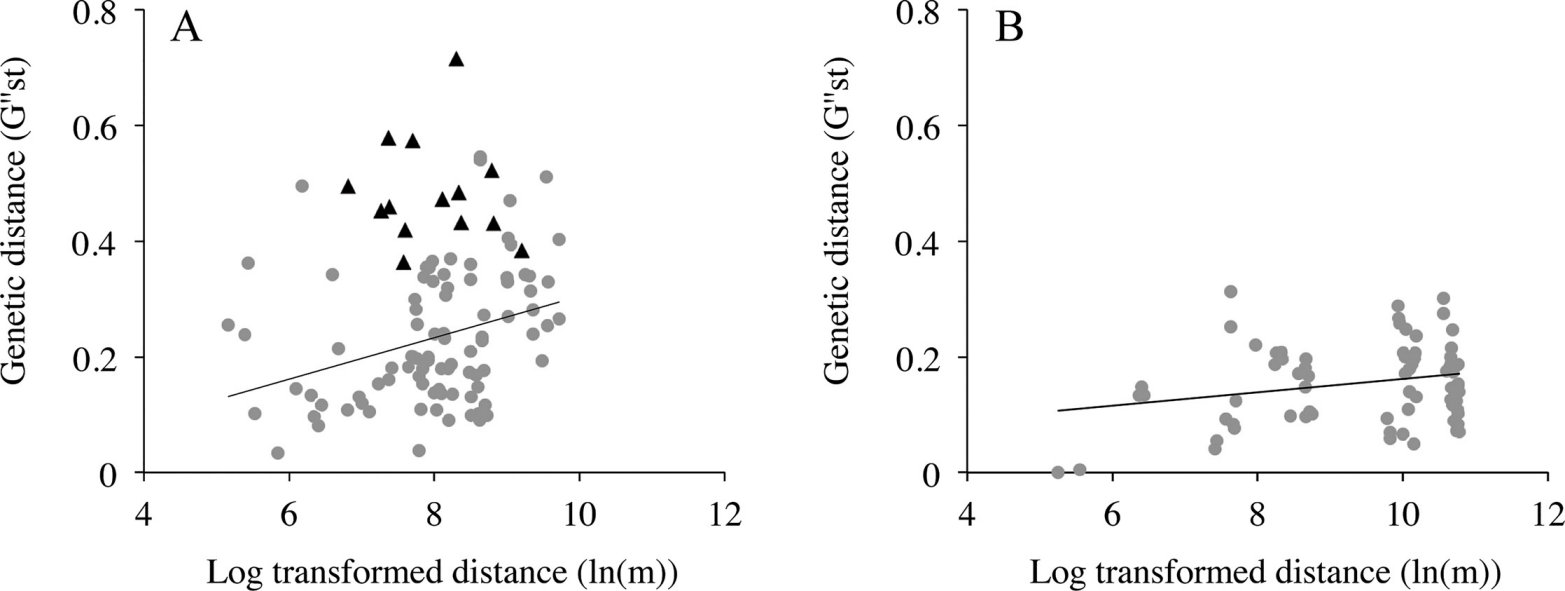
The relationship between pairwise genetic distances (*G”*_ST_) and log transformed geographic distances (ln(m)) for (A) 15 populations of *S*. *granulata* along the Dijle River. Black triangles denote distances from the highly differentiated population ‘Heverlee’ to the other populations and grey circles represent distances between the remaining 14 populations. The result of the mantel test that included population ‘Heverlee’ was not significant, however, we did find a significant isolation-by-distance effect between the other 14 populations (Mantel’s r = 0.327; *P* = 0.048). (B) 13 Populations along the Demer River with a significant isolation-by-distance effect (Mantel’s r = 0.231; *P* = 0.036).

Patterns of spatial autocorrelation were slightly different between the two river systems. Populations along the River Dijle showed no significant isolation-by-distance effect (Mantel’s *r* = 0.229, *P* = 0.179). However, this was caused mainly by the highly differentiated population ‘Heverlee’, which was situated in the middle of the sampling range. When we excluded this population from the analyses we did find a significant isolation-by-distance effect along the Dijle (Mantel’s *r* = 0.327, *P* = 0.048; Fig 2A). Hence, gene flow among populations along the Dijle River takes place generally between populations that are situated close to each other. Along the Demer River we found a lower, yet also significant isolation-by-distance effect (Mantel’s *r* = 0.231, *P* = 0.036; Fig 2B).

**Fig 3:**
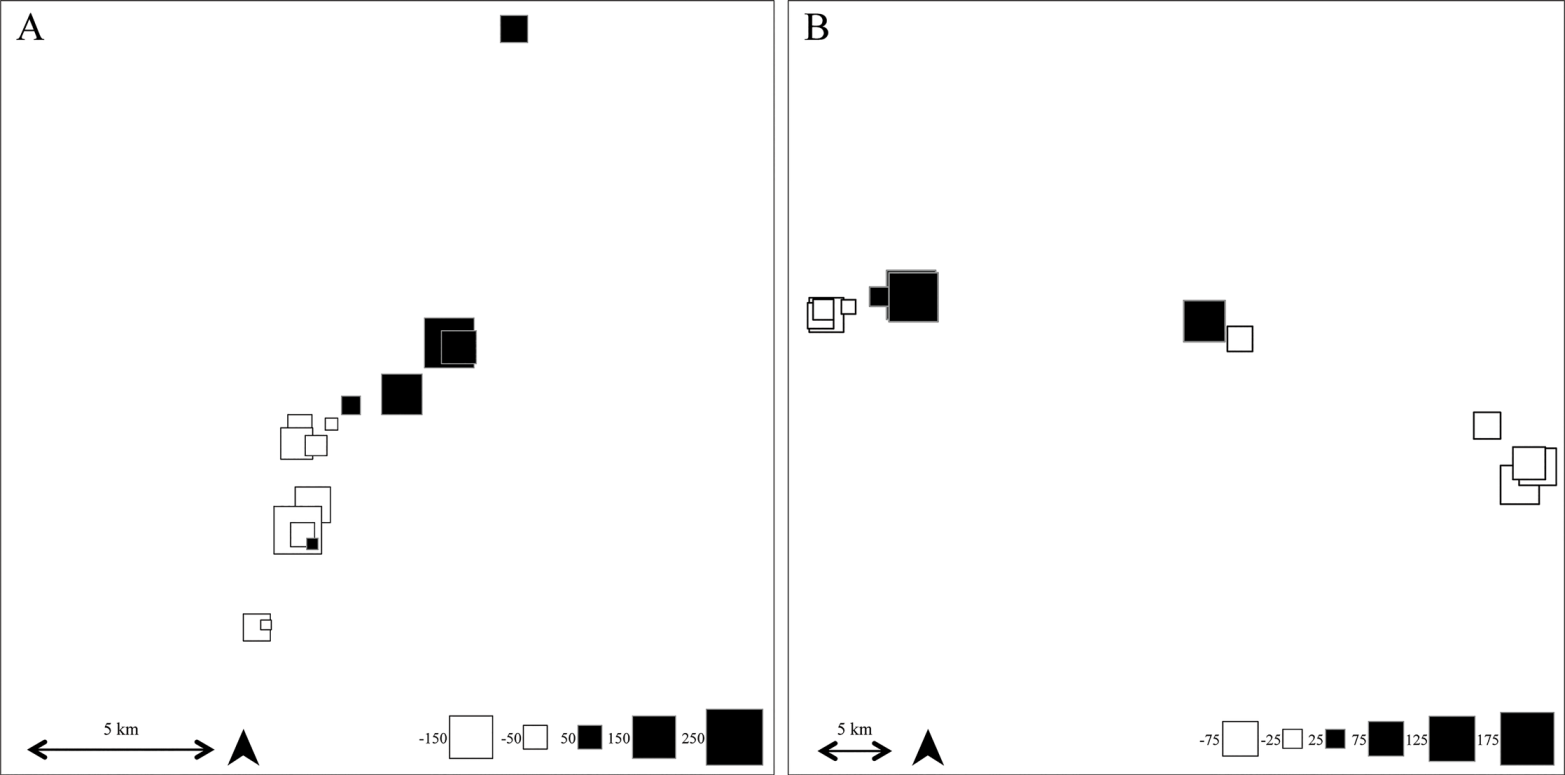
Principal Components of the first axis of the sPCA analysis projected on the spatial structure of the populations along the rivers (A) Dijle and (B) Demer. The size of the squares indicates the size of the PC-value, with black squares representing a positive PC-value and white squares a negative PC-value.

The sPCA results for populations along the river Dijle showed a clear spatial pattern, in which upstream populations were different from downstream populations (Fig 3A). Upstream populations along the Dijle River had negative values on the first principal component axis, while populations downstream had positive principal component values on the first axis. Populations along the Demer River did not show differentiation between upstream and downstream populations. However, a group of populations in the middle of the river was genetically different from populations on either side, upstream and downstream (Fig 3B).

### Population clustering

**Fig 4:**
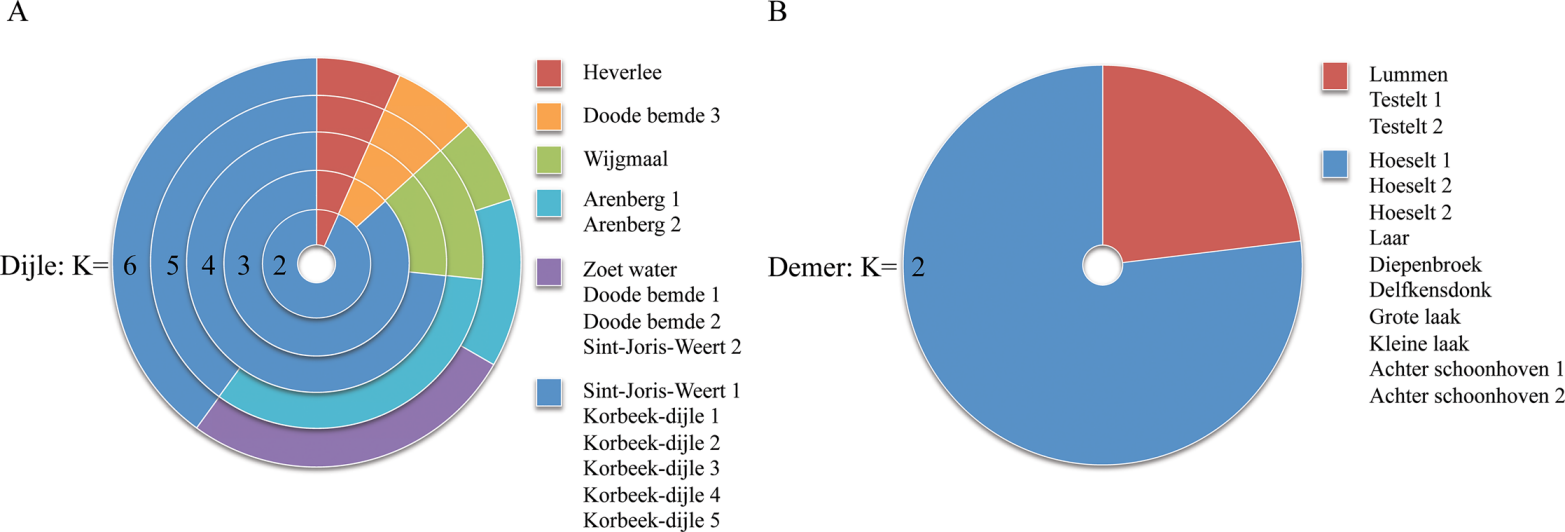
K-means clustering analysis of populations (A) along the Dijle River, showing populations divided into 2–6 clusters. (B) Clusters along the Demer River.

The K-means population clustering analysis showed that populations along the Dijle River grouped to form six separate clusters (Fig 4A). Most clusters are formed between populations that are situated relatively close to each other, illustrating the isolation-by-distance effect, or consisted of one population only. For instance, the highly differentiated population ‘Heverlee’ had a separate cluster for every number of *k*. The K-means cluster analysis of populations along the river Demer showed very similar results as shown by the sPCA analysis and grouped the populations together in two genetic clusters. Populations ‘Testelt 1 and 2’ and ‘Lummen’, situated in the middle of the river's range, were grouped to form one of the two clusters (Fig 4B). These populations in the middle of the Demer River had relatively low allelic diversity (Table 1). As a result they were clustered because these populations were probably missing alleles that were present in all other populations upstream and downstream.

### Unidirectional flow hypothesis

Based on the distribution of genetic diversity along the two rivers, we did not find any indication for an increase in genetic diversity downstream as expected when the river is the main dispersal vector (Table 1). Populations along the Dijle River were not significantly more diverse downstream (*F*_1,13_ = 0.0004, *P* = 0.99) and populations along the river Demer were actually significantly more diverse upstream than downstream (*F*_1,11_ = 5.89, *P* = 0.03).

Moreover, we did not find a relationship between genetic diversity and distance to the river (Dijle: *F*_1,13_ = 0.36, *P* = 0.56; Demer: *F*_1,11_ = 0.0002, *P* = 0.99), indicating that populations closer to the stream did not receive more alleles via the water than populations that were situated further from the river. Finally, genetic diversity was not significantly related to population size (Dijle: *F*_1,13_ = 0.44, *P* = 0.52 Demer: *F*_1,11_ = 1.58, *P* = 0.23; Table 1). Some relatively small populations were genetically very diverse, while several large populations did not show high genetic diversity.

## Discussion

### Genetic structure and among-population differentiation

In this study we investigated the genetic diversity and spatial genetic structure of fragmented and isolated populations of the grassland herb *S*. *granulata* along two river systems. Genetic differentiation of populations along both rivers, based on *G*_ST_, was low in comparison to several other riparian species, such as *Myrica laxiflora* [52], *Silene tatarica* [4] and *Viscaria alpina* [53], but similar to values of population differentiation of other species [6,12,18,54–56]. However, values of *G*_ST_ were potentially underestimated in *S*. *granulata* due to the use of multi-allelic markers and its high ploidy level. *G”*_ST_ and *D*_EST_ values indicated that genetic differentiation between populations of *S*. *granulata* was substantially higher than that based on *G*_ST_.

High population differentiation in combination with a significant isolation-by-distance effect indicates moderate levels of gene flow, primarily between populations that are geographically close to each other. Mantel’s *r* was not very high, suggesting that long-distance seed dispersal and dispersal between non-adjacent populations might take place. Pollen flow could have played an important role in shaping the isolation-by-distance effect since pollen flow mainly occurs between populations that are situated close to each other. Pollen flow across large distances is unlikely given that populations were on average more than 4 km apart along the Dijle and 23 km along the Demer and the species is mainly pollinated by small flies and solitary bees. Seed dispersal probably explains gene flow patterns across larger distances. Gene flow through dispersal of asexually produced bulbils is unlikely since they generally remain attached to the plant until roots have been formed (S. van der Meer, personal observation).

On the other hand, the observed population differentiation patterns might also reflect historic connectivity. A study of the grassland perennial *Succisa pratensis* has shown that current genetic similarity between populations was affected by past connectivity as a result of the long life-span and a persistent seed bank of *S*. *pratensis* [57]. In the past, *S*. *granulata* used to be more abundant in the study area and was not entirely restricted to riparian grasslands, but also occurred in meadows and grasslands of the traditional small-scale agricultural landscape. Current population genetic diversity patterns might reflect historical connectivity, since clonal propagation and polyploidy can buffer loss of genetic diversity.

The observed differences in population differentiation between the two studied rivers were unexpected, since the distances between the populations along the Dijle were smaller, while populations showed higher genetic differentiation. These differences might be explained by a higher population turnover rate along the Dijle. For instance, population ‘Heverlee’ shows signs of a founder event, it has the lowest effective number of alleles but it does not have the lowest number of alleles, suggesting that this population probably had one high frequency allele at most of its loci that were uncommon in other populations. In *Helmholtzia glaberrima* persistent founder effects also increased population differentiation [58].

### Within-population genetic diversity

The studied populations also showed high levels of genetic diversity within populations, which could be explained by the polyploid nature of *S*. *granulata*. Polyploid populations contain more copies of each gene than similar sized diploid populations. Hence, they could be more genetically diverse due to a higher number of mutations and a lower impact of genetic drift [59]. Most other riparian plant species had lower mean values of gene diversity ranging from 0.04 to 0.31 [4,18,52,53,56,58,60,61], while gene diversity ranged from 0.50 up to 0.76 with a mean of 0.68 in the current study. Only seedlings of the riparian tree species *Populus nigra* showed similar high levels of genetic diversity [55]. High genetic diversity is often observed in tree species, which could be explained by selection processes acting at an early stage.

We did not find evidence to support the hypothesis that the river is the main seed dispersal vector, which would lead to accumulation of genetic diversity downstream. In contrast, genetic diversity along the Demer River was higher in populations upstream instead of downstream. Hence, *S*. *granulata* has ways of dispersing seeds, pollen or vegetative structures upstream, like other riparian plant species [11,22,55,58,62–64]. Upstream dispersal of *S*. *granulata* could occur via pollen and seed flow mediated, for instance, by animals, while downstream seed dispersal across large distances could occur during flooding events that inundate the river valleys and riparian grasslands. In *Origanum vulgare*, another inhabitant of riparian grasslands, occasional long distance seed dispersal events could be traced back to extreme-floods of the river Meuse and local colonization was mostly observed after regular flooding events [24]. Thus, even though *S*. *granulata* occurred at a distance from the river, flooding events in the past could have been important for gene flow, shaping the spatial genetic structure of the studied populations.

## Conclusion

Despite the recent decline of *S*. *granulata* in Western Europe, populations along the Dijle and Demer are still genetically diverse. The high ploidy level and longevity of this species may have buffered the negative effects of increasing isolation due to anthropogenic habitat modification. The levels of population differentiation found in this study imply a history of moderate levels of gene flow, mostly between populations that are geographically close to each other. However, these patterns might also reflect historical connectivity when populations were more widespread. In the riparian plant *Myricaria germanica* the historic pattern of gene flow in one of the studied catchments was mainly directed downstream, while contemporary gene flow was bidirectional. This indicates that the importance of individual dispersal vectors (i.e. water, animals, wind) can change over time [64], for instance, due to river confinement. Overall, rivers might play an important role in enabling long-distance seed dispersal during flooding events, but our results suggest that *S*. *granulata* relies on other dispersal vectors that maintain connectivity between increasingly isolated populations.
